# Effectiveness of Exercise Therapy for Postpartum Urinary Incontinence—Systematic Review

**DOI:** 10.3390/jcm15020810

**Published:** 2026-01-19

**Authors:** Maitane Cuesta-Paredes, Noé Labata-Lezaun, Cristina Orts-Ruiz, Carlos López-de-Celis, Elena Estébanez-de-Miguel

**Affiliations:** 1 Department of Physiotherapy, Faculty of Medicine and Health Sciences, Universitat Internacional de Catalunya, 08195 Barcelona, Spain; aqx203217@uic.es; 2 Fisiosan Clinics, 48980 Santurtzi, Spain; 3 Facultad de Ciencias de la Salud, Universidad Vitoria-Gasteiz (EUNEIZ), La Biosfera Ibilbidea, 6, 01013 Vitoria-Gasteiz, Spain; 4 Actium Functional Anatomy Research Group, 08195 Sant Cugat del Vallès, Spain; 5 Physical Therapy Department, Health Sciences Faculty, CEU-Cardenal Herrara University, 03202 Elche, Spain; cristina.orts@uchceu.es; 6 Study Group on Pathology of the Locomotor System in Primary Care (GEPALAP), Institu Universitari d’Investigació en Atenció Primària (IDIAP Jordi Gol), 08007 Barcelona, Spain; 7 Department of Physiatry and Nursing, Health Sciences Faculty, University of Zaragoza, 50009 Zaragoza, Spain; elesteba@unizar.es

**Keywords:** exercise therapy, urinary incontinence, postpartum period

## Abstract

**Background/Objectives**: Urinary incontinence (UI) is a prevalent health condition with a negative impact on quality of life (QoL). Exercise therapy (ET), specifically, pelvic floor muscle training (PFMT), is recommended as a first-line conservative treatment for UI during pregnancy, childbirth, and the postpartum period. This study evaluated the effects of ET on the management of postpartum UI. **Methods**: A systematic search was conducted to identify clinical trials and randomized controlled trials including women over 18 years with postpartum UI. All included studies used ET as the main intervention. Studies were excluded if UI symptoms were attributable to factors outside the urinary tract or if participants had concomitant pathologies. **Results**: From 298 records screened, four trials were included. Three trials reported statistically significant improvements in UI outcomes, while findings for pelvic floor function and QoL showed greater heterogeneity. One trial found that supervised PFMT was associated with greater improvements in urinary symptoms (BFLUTS), vaginal pressure (18.96 mmHg (SD: 9.08)), and endurance (11.32 s (SD: 3.17)) compared to unsupervised training. Another trial using electromyographic biofeedback with electrical stimulation reported a continence rate exceeding 70% on the 20 min pad test, with improvements in perceived burden (VAS), symptoms (UDI), and QoL (IIQ). A third trial combining PFMT with infrared physiotherapy showed improvements in pelvic floor function (PFIQ-7, PFDI-20), urodynamic parameters, urine loss, and QoL (GQOLI-74). In the remaining trial, within-group improvements were observed, with no statistically significant between-group differences. **Conclusions:** ET appears to be beneficial for postpartum UI, with a moderate certainty of evidence. While the greatest benefits are observed with supervised PFMT, the diversity of comparators, and the risk of performance bias limit definitive conclusions regarding its superiority. Given the short-term follow-up, it remains unclear whether the results are influenced by the spontaneous recovery trajectory in the postpartum period and if these effects are sustained in the long term.

## 1. Introduction

According to the International Continence Society (ICS), urinary incontinence (UI) is defined as any involuntary leakage of urine [1]. Since 1978, UI has been recognized as a disease based on its prevalence, impact on quality of life (QoL), and psychosocial consequences [2]. It is estimated that more than 200 million women are affected worldwide [3]. Previous studies have found that the prevalence of any UI ranges from 25% to 45% [2]. Although UI is very common, few people seek medical care for it [4], which suggests a considerable significant underdiagnosis [5].

Pregnancy and childbirth are considered major risk factors for UI, as they are associated with hormonal changes, increased intra-abdominal pressure, and perineal trauma that can reduce pelvic floor muscle (PFM) strength and endurance [6]. A reduction in PFM strength of 22–35% has been reported after pregnancy and childbirth [7]. Postpartum UI persisting beyond three months is associated with a high risk of long-term persistence over the subsequent five years [8]. Other risk factors include maternal age over 35 years, being overweight, and family history [9].

The management of UI is aimed at reducing complications and restoring continence [10,11,12,13]. Clinical guidelines recommend first-line conservative measures, which include the modification of triggering factors, regulation of fluid intake, environmental modifications, the review of pharmacological treatments associated with incontinence, and ET including pelvic floor muscle training (PFMT) [10,11,12,13]. These measures are fundamental to the development of individualized intervention plans and form the basis of the following three main therapeutic approaches: conservative, pharmacological, and surgical [10,11,12,13].

PFMT is considered the first-line conservative treatment, with reported effectiveness rates of 56–75% [14]. PFMT involves repeated contractions of the perineal muscles to improve function and tone [15]. Its effectiveness has been demonstrated when compared to no treatment or placebo, particularly for stress (SUI) and mixed UI, and with no relevant adverse effects [16].

The available evidence indicates that, although PFMT has been shown to be an effective intervention for the treatment of postpartum UI, the heterogeneity of previous studies does not allow for the identification of the most effective training parameters, as follows: frequency, intensity, number of contractions, program duration, type and degree of supervision, body position during exercise, and the potential combination with other interventions such as lumbopelvic stabilization or electrical stimulation (ES) [17,18].

Given the high incidence of UI observed in postpartum women and the methodological heterogeneity among published studies, this systematic review aims to analyze the therapeutic exercise interventions for postpartum UI to identify the training components associated with better clinical outcomes and to assess their impact on QoL.

## 2. Materials and Methods

### 2.1. Design

This systematic review was conducted following the PRISMA 2020 guidelines. The completed PRISMA 2020 checklist is provided in the Supplementary Material (Table S1). The protocol was prospectively registered on the PROSPERO platform with the code CRD420251118045.

### 2.2. Eligibility Criteria

The research question was structured using the PICO framework, considering as population (PP) women over 18 years of age diagnosed with UI during the postpartum period; the intervention (I) consisted of ET programs or PFMT; the comparator (C) included control groups without intervention or with an alternative intervention; and the outcomes (O) focused on the effects of ET on UI, QoL, and applied protocols. The following inclusion criteria were established: (a) original studies such as randomized controlled trials, controlled trials, multicenter studies, pragmatic clinical trials, or pilot trials; and (b) adult women diagnosed with UI during the PP; and (c) studies that used ET as the primary intervention. The exclusion criteria were the following: (a) studies including women with relevant concomitant conditions (prolapse, fecal incontinence, neurological disorders, cognitive impairment, or lack of independent mobility); (b) studies addressing UI in stages other than postpartum; (c) studies aimed at the primary or secondary prevention of UI; and (d) studies without full-text availability.

### 2.3. Search Strategies and Study Selection

Searches were performed in PubMed, PEDro (Physiotherapy Evidence Database), CINAHL ultimate, Scopus and the Cochrane Library for studies published up to 22 July 2025. Mesh terms such as “exercise therapy”, “urinary incontinence”, and “postpartum period” were used, along with keywords such as “pelvic floor dysfunction”, “pelvic floor disorders”, “postnatal”, and “postpartum” combined with the Boolean operators AND and OR. The filters “clinical trial” and “randomized controlled trial” were also applied. The specific search strings for each database are presented in Table 1.

Initially, two reviewers screened titles and abstracts (M.C.P. and C.L.d.C.). In case of discrepancies, a third author (N.L.L.) was consulted. Potentially eligible articles were then subjected to full text review.

### 2.4. Methodological Quality Assessment

Methodological quality was assessed using the PEDro scale, based on the Delphi list developed, consisting of 11 items, of which 10 are considered for the final score (items 2–11) [19]. (Table 2). The assessment was carried out independently by two reviewers, and any discrepancies were resolved by consensus.

Additionally, the Cochrane Collaboration’s Risk of Bias 2 (RoB 2) tool was applied to evaluate the risk of bias in randomized controlled trials. This tool analyzes the following five domains: random sequence generation, deviations from intended interventions, incomplete outcome data, outcome measurement, and selective reporting [23].

The risk of bias in each domain was assessed, classifying it as low risk, high risk, or some concerns, according to the Cochrane Handbook guidelines. Two reviewers independently performed the evaluation, resolving any disagreement through discussion or, if necessary, by consulting a third reviewer.

### 2.5. Data Extraction

Data extraction was performed using a Microsoft Excel database, where relevant information from each included study was recorded. The extracted data comprised the following: (a) author and year of publication; (b) scientific journal and its impact factor; (c) sample size and sample characteristics; (d) type of UI, (e) whether participants were multiparous or primiparous; (f) whether participants had a vaginal birth or a cesarian section; (g) variables related to the training or intervention applied; (h) type of delivery; (i) outcome variables analyzed; (j) measurement instruments used; (k) follow-up duration; and (l) main results reported.

### 2.6. Outcomes

The most commonly analyzed outcome variables in the included studies were (a) UI, considering aspects such as severity, frequency, bladder neck mobility, and urinary symptoms; (b) QoL; and (c) the specific components of the ET programs, such as the training type, dosage, progression, supervision, and complementary modalities.

### 2.7. Data Synthesis and Analysis

A narrative synthesis of the results was performed with the aim of describing and comparing the methodological characteristics, applied interventions, analyzed variables, and reported effects of ET in women with UI during the PP. Extracted information was organized into summary tables to facilitate comparison across studies regarding the type of intervention, duration, sample characteristics, evaluation tools used, and main outcomes. This synthesis facilitated the identification of similarities, differences, and potential response patterns to ET.

Due to the clinical and methodological heterogeneity of the included studies, as well as the limited number of trials (n = 4), a quantitative synthesis (meta-analysis) was not performed. Instead, a narrative synthesis was conducted in accordance with PRISMA guidelines.

### 2.8. Certainty of Evidence (GRADE)

The certainty of evidence was assessed using the Grading of Recommendations Assessment, Development, and Evaluation (GRADE) approach. This assessment was applied to primary outcomes critical for clinical decision making, including UI severity, QoL, and PFM function. In accordance with the GRADE guidelines [24], the certainty of evidence for each outcome was classified as high, moderate, low, or very low.

## 3. Results

A total of 298 articles were identified in the five databases used, as follows: 107 in PubMed, 69 Cochrane, 42 in PEDro, 68 in Scopus, and 12 in CINAHL Ultimate, in total. After removing 126 duplicates, the remaining 172 titles and abstracts were screened, leading to the exclusion of 161 articles. Among the 11 articles reviewed in full text, 1 study was excluded because it did not include the same target population, 6 for using different variables. Finally, four articles were analyzed [17,20,21,22].

**Figure 1:**
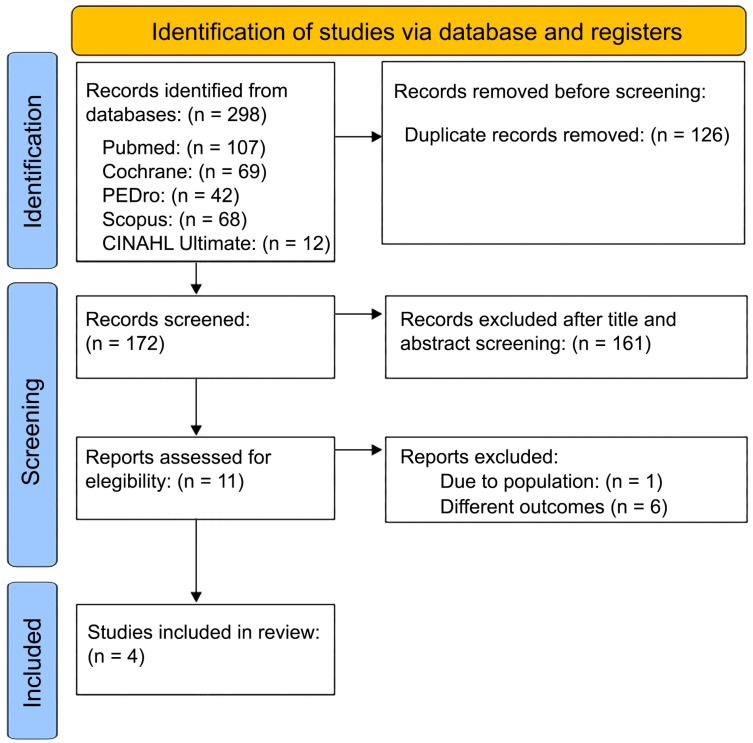
PRISMA flow diagram of study selection.

The process described above regarding the selection of studies is shown in Figure 1 through the flow diagram.

The main findings from the four selected articles are presented below in relation to the variables and research objectives of the present study.

Two of the included studies investigated the effects of PFMT performed with and without professional supervision [20,21]. In both cases, the intervention group (IG) conducted PFMT under the supervision of physiotherapists, while the control group (CG) performed the exercises at home without supervision. The variables assessed included urinary symptoms, using the BFLUTS questionnaire [21], PFM strength and endurance, measured with a perineometer [20,21], and QoL, through self-reported questionnaires [20]. The results indicated statistically significant improvements in the supervised groups (SG) compared to controls in terms of reduction of urinary symptoms, as well as increases in muscle strength and endurance (*p* < 0.05) [21]. In the other study, better outcomes in QoL were also observed, although adherence to the home-based program was variable.

Dumoulin et al. [17] compared two multimodal PFMT programs to a CG that received relaxation massage. The IG performed electrostimulation combined with PFMT using biofeedback, with or without transversus abdominis (TrA) training, in addition to a home-based program. The variables assessed were urinary symptom severity using the 20 min Pad test, perceived burden with the VAS, associated symptoms with the UDI, impact on QoL with the IIQ, and PFM strength using dynamometry. The results showed that more than 70% of women in the IG achieved continence after eight weeks, compared to 0% in the CG, with significant improvements in the Pad test (*p* < 0.001), VAS, UDI, and IIQ (all *p* < 0.002). However, no significant differences were found in PFM strength measured by dynamometry.

In Li & Li [22], the effects of combining PFMT with infrared physiotherapy were evaluated compared to standard postpartum care. The IG received abdominal infrared physiotherapy together with a PFMT program initiated in hospital and continued at home, while the CG received only basic postpartum care guidance and general recommendations on UI. The variables assessed included pelvic floor function using the PFIQ-7 and PFDI-20 questionnaires, urodynamic parameters (PQmax, PVLP, PMUC, and PMU), leakage volume using the Pad test, and QoL through the GQOLI-74. The results demonstrated significant improvements in the IG compared with the CG in muscle strength, urodynamic parameters, reduction of urine leakage, and QoL after two months of treatment.

**Figure 2:**
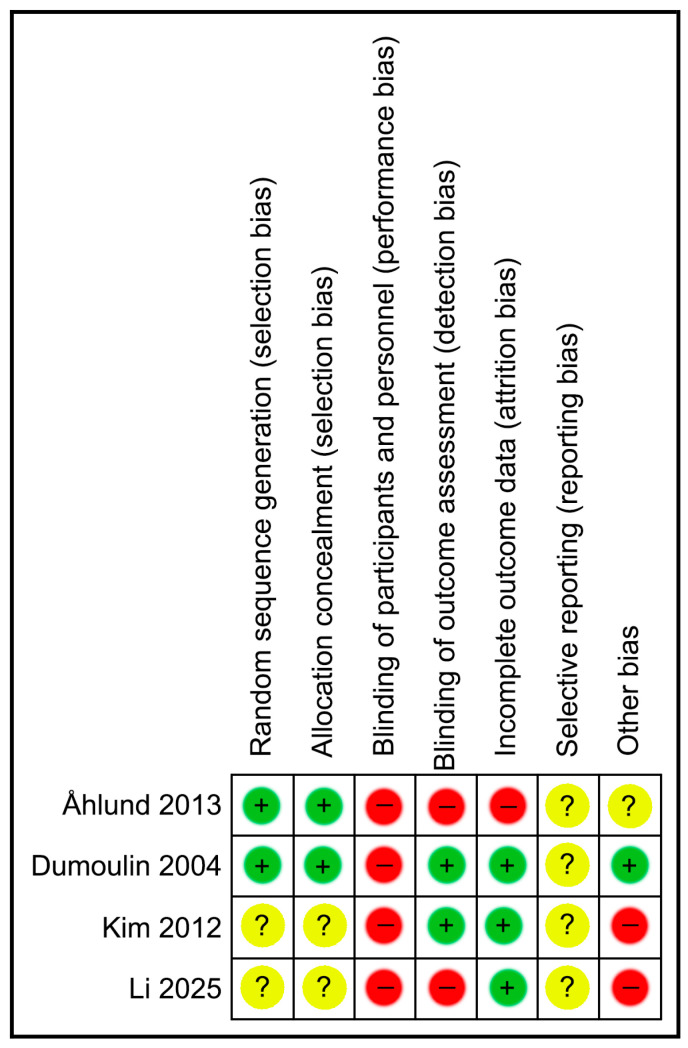
Risk of bias summary. (+): Low risk of bias (green); (−): High risk of bias (red); (?): Unclear risk of bias (yellow) Ahlund et al. [20]; Dumoulin et al. [17]; Kim et al. [21]; Li & Li. [22].

**Figure 3:**
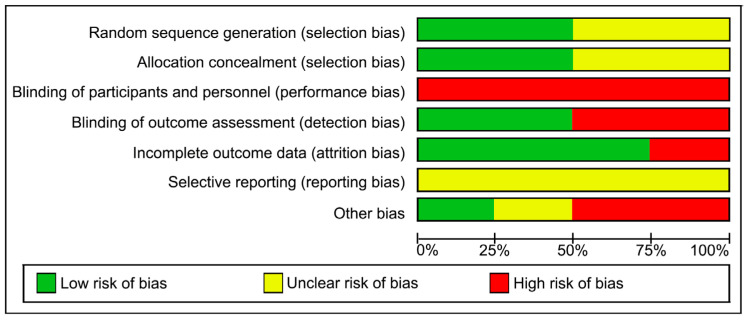
Risk of bias graph.

The methodological quality of the studies was assessed using the PEDro scale and is presented in Table 2. Risk of bias analysis is also presented in Figure 2 and Figure 3. Although PEDro scores ranged from 5 to 8, the risk of bias analysis revealed critical weaknesses in blinding, which limits the certainty of the results.

The certainty of the evidence for the main outcomes was assessed using the GRADE approach and is summarized in Table 3.

For the primary outcome, UI, the certainty of evidence was rated as moderate. Although all included studies were randomized controlled trials, the certainty was downgraded due to methodological limitations, mainly related to lack of participant blinding and incomplete blinding of outcome assessors in some trials, as well as imprecision derived from small sample sizes and short follow-up periods. Nevertheless, most studies consistently reported statistically significant improvements in UI outcomes favoring ET interventions.

For the secondary outcome QoL, the certainty of evidence was rated as low. Downgrading was applied due to risk of bias, imprecision, and heterogeneity in the instruments used to assess QoL, which limited comparability across studies.

The certainty of evidence for PFM function was also rated as low, primarily due to inconsistency in the direction and magnitude of effects across studies, indirectness related to variability in measurement methods, and imprecision associated with small samples and short intervention durations.

The summary of the main characteristics of the studies is presented in Table 4.

## 4. Discussion

The objective of this systematic review was to analyze the available evidence on ET interventions for postpartum UI to identify training components associated with improved clinical outcomes and to evaluate their impact on QoL based on the findings of four randomized controlled trials.

According to the GRADE approach, the certainty of evidence supporting ET for postpartum UI was rated as moderate for UI outcomes and low for QoL and PFM function. Although all included studies were randomized controlled trials, the certainty of evidence was downgraded mainly due to methodological limitations, small sample sizes, and short follow-up durations. These findings suggest that ET may be associated with improvements in postpartum UI; however, further high-quality studies are needed to strengthen confidence in the magnitude and durability of these effects.

In the studies by Åhlund et al. [20] and Kim et al. [21], participants were of similar mean ages, with mean values of 33 years and 31–32 years, respectively. The remaining trials showed a wider age range, from a median of 36–37 years in Dumoulin et al. [17] to a mean age of 26 years in Li & Li [22]. Although differences were observed across studies, all ages fell within the typical reproductive period, and most values aligned with the mean age at first birth in Europe, estimated at 28–30 years [25,26], as well as with the trend toward delayed childbearing described in European demographic literature [27]. In all trials, participants presented UI, with SUI being the predominant subtype in the PP [17,21,22].

Regarding parity, Åhlund et al. [20] included only primiparous women, thereby reducing the potential influence of previous births. This approach is consistent with other reviews that have focused on first-time mothers to reduce obstetric heterogeneity [28,29]. In Li & Li [22], primiparous women accounted for 64–65% of the sample and multiparous women for 34–36%, indicating greater heterogeneity in terms of number of previous births. Dumoulin et al. [17] also included women with different obstetric histories and balanced parity through stratified randomization, whereas Kim et al. [21] recorded parity only as a baseline characteristic. This pattern is similar to that described in European studies such as Manzotti et al. [26], where differences between primiparous and multiparous women are less pronounced within the regional reproductive profile. In contrast, studies included in the synthesis by Zhang et al. [30], conducted in settings with higher multiparity rates, reported samples with a greater number of births per woman, reflecting distinct obstetric profiles.

Regarding mode of delivery, two studies included only women with spontaneous vaginal birth [21,22], following a common criterion that excludes multiple or caesarean deliveries. These exclusions are based on the possibility that such delivery modes involve different pelvic floor injury mechanisms or may require distinct therapeutic approaches. However, this restriction limits the generalizability of findings to the broader postpartum population.

Three trials reported statistically significant improvements during or following the physiotherapy intervention [17,21,22]. One trial reported statistically significant changes in urinary symptoms scores and QoL, together with increases in maximum vaginal squeeze pressure and holding time in the supervised training IG [21]. A second trial showed that more than 70% of women in the IG achieved continence on the pad test during the intervention period, with additional statistically significant improvements in perceived incontinence burden and QoL, whereas no changes were observed in the CG [17]. A third trial found that the combination of PFMT with infrared physiotherapy was associated with improvements in muscle function, urodynamic parameters, reduction in urine loss volume, and QoL compared to the CG [22]. In contrast, the trial by Åhlund et al. [20] did not report statistically significant differences between the IG and CG, although both groups showed improvements in PFM strength and self-reported UI symptoms.

In the study by Dumoulin et al. [17], the achievement of objective continence in over 70% of the IG was not accompanied by significant increases in maximum PFM strength. This finding suggests that clinical recovery may be partly related to improvement in motor coordination and timing of PFM activity during increases in intra-abdominal pressure, rather than muscle hypertrophy alone.

Concerning methodological quality and risk of bias, all four studies were randomized controlled trials [17,20,21]. Each received a score greater than 5 on the PEDro scale, suggesting acceptable methodological quality according to PEDro criteria, despite important limitations identified in the risk-of-bias assessment. The absence of complete blinding, particularly of participants, is a recognized limitation in physiotherapy interventions. Dumoulin et al. [17] and Kim et al. [21] were single-blind studies in which outcome assessors were unaware of group allocation. However, the midwife in Åhlund et al. [20] was not blinded, which may have influenced subjective outcome measures such as the Oxford scale. Li & Li [22] did not report their blinding procedures.

The primary intervention in all studies was ET that included PFMT. PFMT is designed to target the strengthening of slow-twitch muscle fibers to improve tone and support, and fast-twitch fibers to enhance the urethral closure response, both essential for vesico-urethral support and sphincter function. In addition to PFMT, other modalities were examined, including supervised PFMT, PFMT combined with trunk stabilization exercises, biofeedback, ES, and infrared physiotherapy.

Regarding the added value of professional supervision and trunk stabilization, the evidence remains conflicting. While Kim et al. [21] reported that a supervised program incorporating trunk stabilization was associated with statistically significant improvements in selected outcomes compared to unsupervised PFMT. Åhlund et al. [20] found no significant differences between groups, nor any additional benefit from adding stabilization exercises to the standard PFMT protocol. These discrepancies suggest that the specific quality of instruction or the total training dosage may be more influential than the inclusion of complementary exercises.

Biofeedback and ES were used as tools to facilitate awareness and correct muscle contraction technique. In the trial by Dumoulin et al. [17], electromyographic biofeedback was integrated into a supervised multimodal PFMT program. This approach was associated with an objective continence rate exceeding 70% during the intervention period, measured using the 20-min pad test (<2 g urine loss), as well as statistically significant improvements in perceived incontinence burden (VAS), urinary symptoms (UDI), and QoL (IIQ). However, no relevant changes were observed in maximum strength or contraction speed measured by static dynamometry, which were interpreted by the authors as potentially reflecting motor learning processes rather than an actual increase in muscle strength.

In the trial by Kim et al. [21], a perineometer was used as an initial visual biofeedback tool, and statistically significant improvements were reported in urinary symptoms and QoL (BFLUTS). In relation to infrared physiotherapy, the trial by Li & Li [22] evaluated it as a complementary intervention to PFMT. This combination was associated with statistically significant improvements in PFM function, reflected in statistically significant reductions in PFIQ-7 and PFDI-20 scores, as well as statistically significant changes in urodynamic parameters including maximum flow rate (Qmax), Valsalva leak point pressure (VLPP), maximum urethral closure pressure (MUCP), and maximum urethral pressure (MUP). A statistically significantly reduction in urine loss volume in the IG was also observed after one and two months of treatment, together with statistically significant increases in GQOLI-74 scores.

The diversity of outcome measures and assessment scales across the included studies complicates quantitative synthesis and limits cross-study comparability, which is reflected in the lower GRADE ratings assigned to these outcome domains.

This narrative synthesis identified relevant heterogeneity in intervention dosage and session structure, particularly with respect to the number of sets and repetitions, contraction and rest times, and the level of detail with which these parameters were reported.

Regarding session structure, notable differences were observed across the included studies. With respect to the number of sets and repetitions, some protocols prescribed multiple daily sets, as in Åhlund et al. [20] (three sets per day with 8–12 contractions), whereas others implemented structured sessions several times per week, as in Kim et al. [21], resulting in a lower total number of sessions. When reported, the number of repetitions per set ranged between 8 and 12, as described by Åhlund et al. [20], whereas this parameter was not explicitly specified in Dumoulin et al. [17], Kim et al. [21], and Li et al. [22].

Variability was also identified in contraction and rest times. Åhlund et al. [20] described sustained contractions lasting 6–8 s followed by rest periods of similar duration, whereas Dumoulin et al. [17] combined slow and fast contractions without consistently reporting precise activation and relaxation times. In Kim et al. [21] and Li et al. [22], contraction and rest times were not reported in sufficient detail to allow for direct comparison.

Finally, progression of training load (e.g., increases in repetitions, contraction duration, or exercise complexity) was not systematically described in any of the included studies, including Åhlund et al. [20], Dumoulin et al. [17], Kim et al. [21], and Li et al. [22]. This limitation hinders estimation of total training load and complicates comparison of intervention dosage across studies.

In the studies included in this review, the follow-up duration was generally short and heterogeneous, ranging from assessments conducted immediately after the intervention, as in the studies by Dumoulin et al. [17] and Kim et al. [21], which assessed outcomes only after 8 weeks of intervention, to short-term follow-up periods of up to 6 months, as in Åhlund et al. [20]. In the study by Li et al. [22], assessments were performed during the intervention period (at 1 and 2 months), with no subsequent follow-up.

The assessment of outcomes only at the end of the intervention period, without medium- or long-term follow-up, restricts conclusions regarding the sustainability of treatment effects over time. Accordingly, the outcomes observed in the short term may not be representative of long-term continence outcomes.

Given the common occurrence of spontaneous pelvic floor recovery during the first six months postpartum, the findings of the included studies should be interpreted cautiously, as improvements attributed to exercise therapy may partially reflect the natural postpartum recovery trajectory.

### Limitations

The main methodological limitations of the included trials [17,20,21,22] relate to small sample sizes, insufficient blinding, short follow-up periods, and the absence of systematic adherence monitoring. In all studies, limited sample size reduced statistical power and external validity; Dumoulin et al. [17] attributed the lack of significant differences to a Type II error, and Åhlund et al. [20] acknowledged that sample size and loss to follow-up may have influenced the absence of statistical significance in some outcomes. Participant unblinding was a frequent source of bias, as participants were aware of the intervention received, which may have affected subjective outcome measures.

One limitation of this review is that the search was restricted to five databases. Additionally, the included studies lacked long-term follow-up, which limits the assessment of sustained treatment effects. Outcomes were assessed only over follow-up periods ranging from two to six months, which limits the ability to determine the long-term effectiveness and durability of conservative management for postpartum UI. Only the work by Åhlund et al. [20] included an evaluation at six months postpartum; however, this time frame is insufficient to determine the sustainability of the results and the long-term effects of the intervention.

Finally, the absence of standardized adherence-monitoring methods prevented accurate assessment of participants’ compliance, which may have influenced the observed results. Future trials should include larger samples and longer follow-up periods to assess the persistence of treatment effects. The inclusion of more heterogeneous populations, such as multiparous women, women with cesarean delivery, or participants from varied socioeconomic backgrounds, would enhance external validity. Furthermore, examining combinations of PFMT with interventions such as infrared physiotherapy or ES is recommended. Additionally, establishing standardized protocols regarding sets, repetitions, frequency, and intensity to facilitate comparison across studies. Lastly, ensuring assessor blinding and implementing systematic adherence monitoring is crucial for ensuring treatment effectiveness.

## 5. Conclusions

In conclusion, ET including PFMT appears to be a beneficial intervention for reducing postpartum UI symptoms, with a moderate certainty of evidence. However, for other outcomes such as muscle function and QoL, the certainty of the evidence is low due to the imprecision and heterogeneity of the reported results. While supervised programs suggest greater benefits, the diversity of comparators and the high risk of performance bias limit definitive conclusions regarding their superiority over other modalities. Furthermore, most reported improvements are statistical; their clinical relevance remains uncertain, as they have not been calibrated against minimal clinically important differences (MCID). Given that follow-up periods were mostly short-term, it is difficult to distinguish the therapeutic effect of ET from the spontaneous recovery trajectory inherent to the postpartum period. Future research requires larger, methodologically robust RCTs with standardized protocols, long-term follow-ups, and quantitative meta-analyses—including a broader literature search—to conclusively determine the persistence and clinical relevance of these interventions. Despite these limitations, ET including PFMT remains a suitable option for postpartum UI.

## Figures and Tables

**Table 1:** Database and the specific search equations.

Database	Specific Search Equation
PubMed	(exercise therapy [Mesh Terms]) AND ((urinary incontinence [Mesh Terms]) OR (pelvic floor dysfunction) OR (pelvic floor disorder)) AND (postpartum period [Mesh Terms]) AND ((clinical trial [Filter]) OR (randomized controlled trial [Filter]))
PEDro	Abstract & Title: Exercise therapy. Problem: Incontinence. Body part: Perineum or genito-urinary system.
Cochrane	([mh “exercise therapy”] AND ([mh “urinary incontinence”] OR “pelvic floor dysfunction”:ti,ab,kw OR “pelvic floor disorder”:ti,ab,kw) AND ([mh “postpartum period”] OR “postnatal”:ti,ab,kw OR “postpartum”:ti,ab,kw))
CINAHL ultimate	(MH “Therapeutic Exercise+” OR “exercise therapy”) AND (MH “Urinary Incontinence+” OR “pelvic floor dysfunction” OR “pelvic floor disorder”) AND (MH “Postpartum Period” OR “postnatal” OR “postpartum”)
Scopus	(TITLE-ABS-KEY (“exercise therapy” AND (“urinary incontinence” OR “pelvic floor dysfunction” OR “pelvic floor disorder”) AND (“postpartum period” OR “postnatal” OR “postpartum”))) AND (TITLE-ABS-KEY (“randomized controlled trial” OR “clinical trial” OR “randomized” OR “controlled trial”)) AND PUBYEAR < 2026

**Table 2:** PEDro Scale.

	Åhlund et al. 2013 [20]	Dumoulin et al. 2004 [17]	Kim et al. 2012 [21]	Li & Li 2025 [22]
Eligibility criteria *	1	1	1	1
Random allocation	1	1	1	1
Concealed allocation	0	1	0	0
Baseline similarity	1	1	1	1
Blind subjects	0	0	0	0
Blind therapists	0	0	0	0
Blind assessors	1	1	0	0
Less than 15% dropouts	1	1	1	1
Intention-to-treat analysis	1	1	0	0
Between-group comparisons	1	1	1	1
Point measures and variability	1	1	1	1
Total *	7	8	5	5
Average	6.25			

* Item 1 is not used to calculate the PEDro score.

**Table 3:** Summary of findings (GRADE).

Outcome	No of Studies	Study Design	Risk of Bias	Inconsistency	Indirectness	Imprecision	Publication Bias	Certainty of Evidence
UI	4 RCTs	RCT	Serious ^1^	Not serious	Not serious	Serious ^2^	Undetected	MODERATE ⊕⊕⊕◯
QoL	4 RCTs	RCT	Serious ^1^	Not serious	Not serious	Serious ^2^	Undetected	LOW ⊕⊕◯◯
PF muscle function	4 RCTs	RCT	Serious ^1^	Serious ^3^	Serious ^4^	Serious ^2^	Undetected	LOW ⊕⊕◯◯

^1^ Downgraded due to lack of participant and/or assessor blinding in some studies. ^2^ Downgraded due to small sample sizes and short follow-up periods. ^3^ Downgraded due to inconsistent effects across studies. ^4^ Downgraded due to heterogeneity in pelvic floor muscle function measurements. ⊕⊕⊕◯ Moderate certainty; ⊕⊕◯◯ Low certainty.

**Table 4:** Characteristics of the included studies.

Study	Study Design	Population	Methodology (Type of Intervention, Duration, Sets & Repetitions, Frequency, Follow-Up)	Comparison	Outcomes & Measurement Tools	Main Results
Åhlund et al. 2013 [20]	RCT	Primiparous women (18–45 y) with SUI 8 weeks PP. N: 98; IG: 49/CG: 49. BMI; 23 (3.4)/IG: 23 (3.5)/CG: 23 (3.2).	Intervention: Brief 15 min talk. Written PFMT program. Duration: 6 months. Sets & repetitions: 3 fast contractions. 8–12 slow contractions of 6”. Frequency: 7 days/week. Follow-up: 6 months from baseline.	Digital palpation. General PP advice in written form.	UI symptoms: ICIQ-FLUTS. PFM strength: Perineometer/Oxford scale. PFM endurance: Perineometer (s).	Both groups improved in MVC and muscle endurance (*p* < 0.05); the IG increased MVC from 16.2 to 26.0 cmHg and endurance from 9.6 to 26.7 s, while the CG improved from 12.1 to 18.2 cmHg and from 12.0 to 23.4 s. No between-group differences were found in MVC, endurance, or Oxford Scale scores. Both groups showed improvements in ICIQ-FLUTS frequency and incontinence scores, while voiding improved only in the CG. The intervention was effective, but home-based PFMT with written instructions was as effective as supervised follow-up every six weeks.
Dumoulin et al. 2004 [17]	RCT	Women (<45 y) with persistent SUI for 3 months or more PP. N: 64; EMSP-G: 21/EMSP + TrA-G: 23/GC:20. BMI; EMSP-G: 24.20 (22.83–26.19)/EMSP + TrA-G: 22.17 (20.62–24.15)/PFMT-G: 24.32 (21.92–26.07).	Intervention: PFMT-G: ES + supervised PFMT with BFB + home program. PFMT + TrA-G: ES + PFMT with BFB + 30’supervised TrA training + home program. Duration: 8 weeks. Sets & repetitions: PFMT-G: 15’ ES with PFM + 25’ PFMT with BFB. PFMT + TrA-G: same as PFMT group + 30’ TrA training. Frequency: Supervised intervention: 1/week. Home program: 5 days/week. Follow-up: 8 weeks.	8 weekly sessions of relaxing back and limb massages. They were asked not to perform PFMT at home.	UI symptoms: 20-min Pad test. Associated UI symptoms: UDI. QoL: IIQ. Perceived burden of UI: VAS.	Both treatment groups improved significantly in the Pad test (*p* < 0.001), whereas the CG showed no improvement (*p* > 0.243). More than 70% of women in the PFMT and PFMT + TrA groups achieved objective continence (<2 g), compared with 0% in the CG; approximately 90% reduced urine loss by >50%, versus 10% in the CG. Both treatment groups also improved in VAS, UDI and IIQ scores (all *p* < 0.002), with no changes in the CG. No differences were found in maximal PFM strength or contraction speed across the three groups. There were no between-group differences between PFMT and PFMT + TrA, indicating that adding abdominal training did not enhance outcomes. The intervention was effective and showed high adherence, with only 6% dropout.
Kim et al. 2012 [21]	RCT	Women (28–35 y) with SUI PP. N: 20; IG: 10/CG: 10. BMI; IG: 23.58 (1.79)/CG: 24.61 (1.82).	Intervention: PFMT + trunk stabilization + supervision. Support with verbal instructions and digital palpation. Duration: 8 weeks. Sets & repetitions: 23 sessions of 1 h. Frequency: 3 days/week. Follow-up: 8 weeks.	PFMT + trunk stabilization without supervision.	Severity of UI symptoms: BFLUTS. PFM strength: Perineometer (Kontinence Clinical HMT 2000, Seoul, Korea).	Both groups improved in perineometer outcomes, but the SG showed significantly greater gains in maximal vaginal squeeze pressure Δ18.96 ± 9.08 mmHg vs. Δ2.67 ± 3.64 mmHg, *p* < 0.05) and hold time (Δ11.32 ± 3.17 s vs. Δ5.72 ± 2.29 s, *p* < 0.05). Significant pre-post improvements were observed in the SG for both measures (*p* < 0.01), while the unsupervised group improved only in hold time. The SG also demonstrated larger reductions in BFLUTS urinary symptom scores (Δ−27.22 ± 6.20 vs. Δ−18.22 ± 5.49) and QoL scores (Δ−5.33 ± 2.96 vs. Δ−1.78 ± 3.93), as well as in total BFLUTS score (Δ−32.56 ± 8.17 vs. Δ−20.00 ± 6.67) (all *p* < 0.05). Pre-post differences were significant across all BFLUTS subdomains in the SG (*p* < 0.01), whereas the unsupervised group showed significant changes only in urinary symptoms and total score. Supervised PFMT with Trunk stabilization was associated with greater statistically significant improvements than the unsupervised program.
Li & Li, 2025 [22]	RCT	Women (≥18 y) with PPUI. N: 102; GI: 50/GC: 52.	Intervention: IRT + PFMT. Duration: 2 months. Sets & repetitions: IRT: 20 sessions of 30’. PFMT: 15’. Frequency: IRT: 2 times/day during 10 days. PFMT: 3 times/ day. Follow-up: 2 months.	General PP advice in written form.	UI symptoms: ICIQ-FLUTS. PFM strength: Perineometer/Oxford scale. PFM endurance: Perineometer (s). QoL: GQOLI-74. Associated UI symptoms: PFDI-20.	Both groups improved in pelvic floor function, urodynamic measures, leakage volume, and QoL, however, the IG showed greater gains. The IG had larger reductions in PFIQ-7 and PFDI-20 scores compared with the CG (*p* < 0.05), greater increases in urodynamic parameters such as PVLP (*p* < 0.001), and a significantly lower leakage volume at both 1 and 2 months (*p* < 0.001). QoL scores (GQOLI-74) improved to a greater extent in the IG across all subdomains (*p* < 0.003). Pelvic floor rehabilitation indicators (vaginal muscle tension, muscle voltage, and nocturia frequency) also showed significantly larger improvements in the IG compared with the CG (*p* < 0.022). Overall, the combined intervention was more effective than conventional postpartum care in improving pelvic floor function, urodynamic outcomes, leakage volume, and QoL.

RCT, randomized controlled trial; R, rest; SUI, stress urinary incontinence; PP, postpartum period; BMI, body mass index; BFB, biofeedback; IG, Intervention Group; CG, Control Group; G, Group; SG, Supervised Group; PFMT, pelvic floor muscle training; TrA, transversus abdominis; ES, electrical stimulation; IU, urinary incontinence; PFM, pelvic floor muscle; ICIQ-UI, International Consultation on Incontinence Questionnaire—Urinary Incontinence; ICIQ-LUTS, International Consultation on Incontinence Questionnaire—Female Lower Urinary Tract Symptoms; IRT: infrared therapy; QoL, quality of life; IIQ, Incontinence Impact Questionnaire; VAS, Visual Analog Scale; UDI, Urogenital Distress Inventory; BFLUTS, Bristol Female Lower Urinary Tract Symptoms questionnaire; GQOLI-74, Generic Quality of Life Inventory-74; PFDI-20, Pelvic Floor Distress Inventory-20.

## Data Availability

No new data were created or analyzed in this study.

## Supplementary Materials

The following supporting information can be downloaded at: https://www.mdpi.com/article/10.3390/jcm15020810/s1, Table S1: PRISMA checklist.

## Abbreviations

The following abbreviations are used in this manuscript:

RCT	Randomized Controlled Trial
R	Rest
SUI	Stress Urinary Incontinence
PP	Postpartum Period
IG	Intervention Group
CG	Control Group
G	Group
SG	Supervised Group
PFMT	Pelvic Floor Muscle Training
TrA	Transversus Abdominis
ES	Electrical Stimulation
UI	Urinary Incontinence
PFM	Pelvic Floor Muscle
ICIQ-UI	International Consultation on Incontinence Questionnaire—Urinary Incontinence
ICIQ-LUTS	International Consultation on Incontinence Questionnaire—Female Lower Urinary Tract Symptoms
QoL	Quality of Life
UDI	Urogenital Distress Inventory
IIQ	Incontinence Impact Questionnaire
VAS	Visual Analog Scale
BFLUTS	Bristol Female Lower Urinary Tract Symptoms questionnaire
GQOLI-74	Generic Quality of Life Inventory -74
PFDI	Pelvic Floor Distress Inventory
MVC	Maximum Voluntary Contraction
ET	Exercise Therapy
IRT	Infrared Therapy
BMI	Body Mass Index
